# Evolutionary stasis: creationism, evolution and climate change in the Accelerated Christian Education curriculum

**DOI:** 10.1007/s11422-023-10187-y

**Published:** 2023-05-18

**Authors:** Jenna Scaramanga, Michael J. Reiss

**Affiliations:** University College London, 20 Bedford Way, London, WC1H 0AL UK

**Keywords:** Creationism, Curriculum, Evolution, Textbook analysis, Workbooks

## Abstract

There has been little consideration in the science education literature of schools or curricula that advocate creationism. Accelerated Christian Education (ACE) is among the world’s largest providers of creationist science materials with a curriculum divided into a system of workbooks which students complete at their own speed. This article examines the ways in which ACE presents particular areas of science that it considers to be contentious, namely evolution and climate change. The ACE curriculum has recently been rewritten, and we show that, like previous editions, the current curriculum relies on rote memorisation to the exclusion of other styles of learning, and that information presented is often misleading or distorted. Religious explanations of natural phenomena are sometimes given in place of scientific ones, and creationist assumptions are inserted into lessons not directly related to evolution or the Big Bang. Those who reject creationism are depicted as making an immoral choice. ACE’s recent curricula also add material denying the role of humans in climate change. It is argued that both the teaching methods and content of the ACE curriculum place students at an educational disadvantage.

Creationism exists in a number of different versions, but in some countries, a high proportion of adults reject the theory of evolution (Miller, Scott and Okamoto 2006). Instead, they believe that the Earth came into existence as described by a literal (fundamentalist) reading of the early parts of the Bible, the Qu’ran or other scriptures and that the most that evolution has done is to change species into closely related species. Quantitative international comparisons are difficult due to variations in the precise questions asked and the fact that few studies have been undertaken in countries with very high levels of religious observance. In the US, about 35% of the public rejects the theory of evolution (Miller, Scott, Ackerman, Laspra, Branch, Polino and Huffaker 2022).

Although responses to creationism featured sporadically in the science education literature in the twentieth century, in the US, much of it was seen through the lens of the (in) famous 1925 Scopes Trial (Laats 2021). It is only more recently that a significant amount of attention has been paid to creationism by science educators as opposed to historians. Creationism is widespread, and there are indications that there are more countries in which schools are becoming battlegrounds for the issue. For example while the USA has had several decades of legal battles about the place of creationism (Moore 2007), school-based conflicts over the issue have become more frequent in a number of other countries too (Blancke, Hjermitslev and Kjærgaard 2014).

Perhaps unsurprisingly, the emphasis in the science education literature when considering creationism has been on how schools, colleges and other sites of learning (e.g. museums) might help students from creationist backgrounds learn about and/or accept the theory of evolution (e.g. Reiss 2011). In addition, there is a literature about schools that intentionally teach creationism. Back in 1980, Nordin and Turner (1980) pointed out that the fastest growing section of the US school education was that of private Protestant fundamentalist schools, and a suite of studies, many of them book length, followed. One of the best known is the 1986 book *God’s choice: The total world of a fundamentalist Christian school*, in which Peshkin, a professor of education, drew on 18 months’ experience within such a school. Peshkin’s focus was on the relationship between religious doctrine and educational practice. Other studies include Susan Rose’s (1988) *Keeping them out of the hands of Satan: Evangelical schooling in America* and Peter Lewis’ 1991 PhD dissertation on alternative schools and Christian fundamentalist schools. Recently, Guhin (2016; 2021) has written about how both Muslim and evangelical Protestant schools define, through boundary work, what is essential to religious identity and what (in an Aristotelian sense) is accidental.

It is in the interests of science teachers, science education researchers, science communicators and education policymakers to keep abreast of developments in creationist education. Awareness of the content of creationist science curricula can help lecturers and admissions tutors at colleges and universities, who need to be aware of misconceptions and gaps in knowledge that creationist students may have (Blancke, Boudry, Braeckman, De Smedt and De Cruz 2011), as well as the reasons they may be resistant to evolutionary theory (Harms and Reiss 2019). School students who have been taught using creationist curricula sometimes transfer into mainstream schools, and teachers may need to give them additional support (cf. Carins 2002; Baumgardt 2006). Those who campaign for evolution education in schools should also remember that today’s creationist students are tomorrow’s textbook campaigners.

Accordingly, this article examines the ways in which Accelerated Christian Education (ACE), possibly the largest global provider of creationist education, presents particular, contentious areas of science. Our specific research question is: ‘How does Accelerated Christian Education present creationism, evolution and climate change in its educational materials?’

## Literature review

There is a large literature on the difficulties that school students and people in general have in understanding and accepting the theory of evolution. These difficulties can be divided into three categories: one to do with the conceptual demands of evolution (Stern, Kampourakis, Huneault, Silveira and Müller 2018), one to do with a perceived clash between religion and evolution (Long 2011) and (a much smaller literature) one to do with the existential concerns that evolution can raise—issues to do with such matters as death, extinction and whether life is meaningless (Newall 2021).

One of the arguments raised by creationists against the teaching of evolution in school science lessons is that it is not even a scientific theory, partly on the grounds that, having (supposedly) taken place in the past, much of evolution is not amenable to scientific testing—at the same time holding that when evolutionary predictions can be tested, they are found to be wanting (discussed by Reiss 2018). Unsurprisingly, both scientists and science educators have robustly defended evolution against these charges—see almost any article in the journal *Evolution: Education and Outreach* as well as texts on the nature (Abd-El-Khalick and Lederman 2000; Lombrozo, Thanukos and Weisberg 2008) or features (Matthews 2012) of science.

Accelerated Christian Education (ACE) is possibly the largest global supplier of creationist education, principally in English though materials exist in Afrikaans and Spanish. It was funded by Donald and Esther Howard in 1970 and produces materials for use across K-12 (in the USA and elsewhere) in mathematics, English, literature and creative writing, word building/etymology, science, social studies and Old and New Testament. The three largest publishers of creationist science curricula for use in Christian schools and home schools are ACE, BJU Press and Abeka. All are theologically similar, sharing roots at Bob Jones University (Laats 2010). While BJU Press and Abeka appear to be more widely used in the USA (Klein 2021), they do not claim, as ACE recently has, to be used in schools in more than 140 countries (ACE 2020a). Because of their resistance to official registration, confirming numbers is difficult, but at least one-third (2400 in total) of schools in the USA participating in ‘school choice’ voucher programmes use one of these curricula (Klein 2017). Answers in Genesis is a more famous creationist organisation, but it does not produce a comprehensive science curriculum. Children’s exposure to Answers in Genesis is likely to be extracurricular, whereas ACE is probably the principal or sole source of formal science education for many students in Christian schools, particularly in the USA. Creationism has long been central to ACE’s mission. As long as 30 years ago, its vice president presented a paper at the First International Conference on Creationism, titled ‘Perpetuation of Creationism Through Theistic Education’. That paper equated teaching evolution with atheism. Together, it argued, evolution and atheism ‘perpetuate society governed by criminal elements and will sound the death knell of society governed by law’ (Johnson 1986, p. 165).

ACE has been criticised by Christians (Hill 1990; R. Hunter 1985; Moser and Mueller 1980), by government education departments (Alberta Department of Education 1985; Beeke 1992; R. Hunter 1985), by independent academics (Berliner 1997; Fleming and Hunt 1987; Speck and Prideaux 1993) and by news media (Katz 2014; Loxton 2012; Shaw 2009). One review of the ACE curriculum has judged it unacceptable on grounds of intolerance:

The unacceptable ratings were given because of the repeated condemnation of those who reject the author's interpretations of the Bible as these pertain to science. Those who challenge the explanations given in PACEs [explained below] … to historical events and scientific phenomena are described as being ‘godless’, ‘anti-biblical’, ‘foolish’ and ‘a fake teacher’.

Paterson (2003) takes a similar view. Independent academic reviews have argued that ACE’s individualised, workbook-based method is unsuitable for science teaching:

There is no room within this method of learning for the negotiation of topics, for whole class problem-solving, for the generation of ideas, for the formulating and testing of hypotheses, discussion of results and social application …

The development of science skills, the powers of critical thought, and basic scientific literacy and numeracy are important emphases in current science curricula. However the reading comprehension mode of ACE science denies the process approach to science curriculum and the allied problem-solving approach to the teaching of science skills. The discipline of science cannot be learnt in carrels. There are science skills which can be developed only in the interactive teaching mode. Although some ACE schools may add practicals to PACE work, these are inadequate as a method of teaching the skills of science as they are not integrated fully into the process of teaching and learning science. The PACEs themselves do not foster the skills of science (Alberta Department of Education 1985, p. 24).

(Speck and Prideaux 1993, pp. 290–291)

## Methods

ACE is currently in the process of releasing a new, fourth edition of its curriculum. This is a relatively rare event. The third edition of its science curriculum was released gradually, with the first grade introduced in 1977 but the eleventh grade not completed until 1995 (the twelfth grade, oddly, arrived in 1987). While there were some revisions to the third edition during its lifespan, changes to the text were minimal (Scaramanga 2017, p. 52). The fourth edition of first grade science was released in 2009. Subsequent grades have followed gradually, with the eighth grade completed in 2016 and ninth grade released in 2020. This makes now a good time to consider how a major form of creationist education looks for the next generation of students.

The ACE curriculum consists of a series of self-instructional workbooks, called PACEs (Packets of Accelerated Christian Education). Twelve PACEs per academic subject make up one grade’s worth of study. Students entering the curriculum complete a diagnostic test to determine, where in this series, they should start. They then progress through the PACEs at their own speed until graduation. PACEs are self-contained and include everything the student needs to know in order to pass the tests. In higher science grades, students watch DVDs of laboratory experiments, which is said to make up for the lack of practical activities. Students complete their PACE work in ‘offices’ (learning carrels), which are desks facing the wall, separated from their neighbours by vertical dividers. If students need help from staff, they raise a flag. Otherwise, PACE work is completed alone and in silence (ACE 2010a, 2012).

There is a long tradition in educational research of analysing science and other subject curricula and teaching materials from a range of perspectives and in a variety of ways (e.g. Beyer, Delgado, Davis, and Krajcik 2009; Hickman and Porfilio 2012; Laats 2016). The analytical approach we employed uses a version of qualitative content analysis (Drisko and Maschis 2015). The PACEs were read word for word by the first author (a former ACE student whose doctorate researched student experiences at ACE schools), with examples that were judged to be open to more than one reading discussed with the second author (a science educator and author of school science textbooks and other materials with a doctorate in evolutionary biology, and who supervised the first author’s doctorate) until agreement was reached. We paid particular attention to wordings and visual representations that alluded, expressly or otherwise, to creationism, evolution and (for reasons we explain below) climate change. As is typical for a number of versions of qualitative content analysis (Hsieh and Shannon 2005), no formal coding was undertaken. As will be evident below, we are more interested in building an argument as to how science educators are likely to react on reading the PACEs than in drawing any conclusions from conventional coding. Nor, although the material was read critically, was there a need for the formal use of critical discourse analysis (e.g. Fairclough 2003); the attention to documentation typical of historical enquiry (e.g. McCulloch 2004) sufficed because we were mainly interested in how the materials were likely to be read by those for whom they had been written.

This article, therefore, principally considers what ACE currently (in its fourth edition) teaches about creationism, evolution and climate change. We also discuss how much the fourth has changed from the third edition, by comparing one quarter of the second, fourth and eighth and ninth grade science PACEs from the third and fourth editions (Table 1). The sample chosen for comparison of the second and fourth grades was opportunistic, based on which obsolete PACEs could be obtained. We had access to all of the eighth and ninth grade third edition PACEs, so we selected for comparison those with the strongest emphasis on creationism. These grades were chosen because at the time of analysis, all existed in a fourth edition, and they are intended for a range of ages. We sampled a quarter of the science PACEs because the volume of material is substantial (there are 12 PACEs per grade level, and each PACE typically runs to about 50 pages).

**Table 1:** PACEs analysed and reasons for inclusion

Grade	Relevant stated learning outcomes (from ACE 2019, pp. 18–20)
Second	The student… **Expands his knowledge** of the days of Creation, the first man and woman Learns how God made every person **unique** through the introduction of fingerprints, etc.
Fourth	The student… Learns about the **water cycle** [Noah’s Flood provided as an explanation] *Not listed as an objective, but included: Scientific method*
Eighth (Earth Science)	The student… **Explores** the wonders, resources, and cycles of God’s Creation Searches **proofs** of Creation and the Flood
Ninth (Biology)	The student… Views the wonders of the Creator as he studies the **structure** and **function** of man’s skin, skeleton, and muscles Observes **scientific proof** for Creation of fish, amphibians, reptiles, and invertebrates

## Results

Our analysis reveals both the way in evolution and climate change are presented (or not) in ACE materials and the extent to which such presentations have changed from the third to the fourth edition of the PACEs. Initially, we present our findings by grade level as this reflects the order in which students complete their PACEs, but subsequently, we make more general points that cut across particular grades.

### Second grade

A recurrent activity in the second grade PACEs is memorising what God made on each of the 6 days of creation. Online previews of the PACEs reveal that this activity is also a feature of the first and third grades (ChristianBook.com 2021a, 2021b). In this respect, they are unchanged from the third edition (Johnson 1986, p. 164). There are regular mentions of God in what might otherwise be taken to be scientific content. The reader is encouraged to thank God for his creation—‘Thank you God for these heavenly bodies above’ (ACE 2010b, p. 23); scientific discussions are prefaced with a reminder that God made the object in question—‘Since God made the sun, people are able to live on the Earth’ (ACE 2010b, p. 13)—or God is presented as an explanation for how biological processes work:

Fish are able to hear because sounds travel to them through water. God gave fish a way to hear sounds in the water. Fish can hear the sound of my footsteps and quickly swim away. God helps fish hear and feel sounds in the water.

(ACE 2010c, p. 15)

Student activities consist either of matching images with words, or fill-in-the-blank items, often with multiple choice options:

God gives the fish what it ________ (seeds, needs, heeds)

Who gives the fish what it needs? _______ (Mother, God, Father)

(ACE 2010c, p. 22)

It is a fundamental ACE belief that students should be shielded from non-Christian ideas and beliefs (ACE 2021). Accordingly, there is no indication at this level that creationism is at all disputed. Based on the available information, students could only conclude that the *Genesis* creation accounts are literally true, scientific and universally accepted.

### Fourth grade

At this level, the scientific information is presented in the form of stories. In the following extract, students read about a trip to a science museum, where a tour guide tells them:

Knowing what God says about Creation and the Flood helps me understand more about geology … The Bible and true science are not different in what they say about the earth … we can learn about geology from the Bible.

(ACE 2010g, p. 17)

The students’ teacher says:

At the museum this afternoon, we will learn about some special men who used the knowledge God gave them to discover more about our wonderful world … As they worked hard and carefully observed the world around them, God gave them the answers to their questions.

(ACE 2010g, p. 6)

It appears that faith in God is closely linked with, if not essential for, the ability to conduct science. The Christian beliefs of Matthew Maury (1806–1873, US oceanographer and meteorologist), Isaac Newton and Robert Boyle are strongly emphasised. Newton ‘studied the Word of God, and God helped him become a great scientist’ (Ibid, p. 31), while Boyle was able to formulate his eponymous law ‘[w]ith his knowledge of God’s Word and through testing of his ideas’ (Ibid, p. 32). One might reasonably conclude from the text that successful scientific endeavour is dependent on conservative Christian faith. Indeed, ACE has more explicitly stated this view elsewhere (ACE 2011, pp. 8, 24; Johnson 1986, p. 163).

Once again, material not directly related to creationism is given a creationist slant, such as the study of the human heart: ‘God put a special muscle inside your body to pump in fresh air with oxygen in it’ (ACE 2010d, p. 10). The lungs are discussed similarly.

It remains the case in the fourth grade that students are not exposed to any ideas contrary to ACE’s literal interpretation of the Bible. Here, however, the text lays the groundwork for the rejection of evolution:

An idea is not a scientific fact if it cannot be observed and proven to be correct by experiments … If a man says that something is true, but no one has seen it happen, then it is only an idea. It is not a scientific fact. The only things that do not have to be proven by experiments are the things God tells us in His Word, the Holy Bible.

(ACE 2010g, p. 18)

By defining science narrowly as that which can be seen directly through observation and confirmed though experimentation, the text prepares students to see evolution, cosmology and climate change as unscientific. Science is also depicted as definitively ‘proving’ or ‘disproving’ hypotheses, so that it manifests objective truth. The text seems to rule out more nuanced understandings of science as offering a provisional best explanation. Instead, its definitions are designed to insulate students against ideas ACE finds unacceptable. It continues:

We cannot always believe the ideas people have because their reasoning may be wrong ...

If a person says something that does not agree with what God has said in the Holy Bible, then we know the person is wrong. Though others may agree with the person, that does not make his idea true.

Scientists can and do make mistakes. Some science books have mistakes because people have written them, and people make mistakes. However, there are no mistakes in God’s Book, the Holy Bible.

(ACE 2010g, p. 19)

Creationist notions continue to crop up in what some might consider to be unlikely places. The study of scientific measurement begins with Noah’s Ark, of which we learn: ‘We do not know exactly how many animals were on the Ark … but we do know God told Noah exactly how big the Ark needed to be. With three decks, or floors, it was big enough for all the animals and Noah’s family’ (ACE 2010f, p. 7).

Past reviews of the ACE curriculum have criticised it not just for its content but also for the low-level cognitive tasks, use of rote memorisation and the sometimes meaningless multiple choice options provided (Berliner 1997; Speck and Prideaux 1993). Little has improved in this regard:

Sister means (a) a daughter of one’s parents (b) a pretty pony

(ACE 2010e, p. 5)

Deck means (a) an angry elephant (b) a floor of a boat

(ACE, 2010f, p. 4)

### Eighth grade

The eighth grade is the level at which ACE promotes creationism most strongly (Johnson 1986). The PACEs examined here were chosen because of their creationist content in the third edition. *Science 1086* (ACE 1986a) discussed Noah’s Ark; *1089* (ACE 1986b) was one of several PACEs to advance the idea that prior to Noah’s Flood the Earth was surrounded by a vapour canopy (the claimed source of the floodwaters); and *1096* (ACE 1986c) was devoted entirely to creationist arguments.

The third edition of *Science 1086*, like many third edition PACEs, contained factual errors not obviously connected to creationism (D. Hunter 2014a, 2014b, 2014c). Those cited by Hunter are corrected in the fourth edition. The focus on Noah’s Flood has also been reduced; it is now mentioned 15 times on 8 pages, compared with 26 mentions on 11 pages in the third edition. It is in this PACE, however, that students read their first explicit acknowledgement that not everyone is a creationist: ‘Some scientists reject the Bible and the truth of a young created Earth’ (ACE 2016a, p. 24). The following paragraphs make it sound as if geological evidence unequivocally rules out an old Earth, pointing to the Lewis Overthrust in Glacier National Park as evidence (though it is not referred to by name in the text). This claim appeared in the first modern ‘creation science’ text, *The Genesis Flood* (Whitcomb and Morris 1961). Other young Earth arguments advanced here are little newer.

The inclusion of the vapour canopy claim in ACE’s third edition was particularly interesting. The canopy was postulated as an attempt to explain scientifically the waters ‘above the firmament’ in *Genesis* 1:7, and as a source of the Noahic floodwaters. In the year, the eighth grade PACEs were released, a paper at the First International Conference on Creationism admitted that the canopy appeared hard to defend because it would lead to an intolerable temperature on Earth’s surface (Vardiman 1986). We know ACE’s Vice President was in attendance, since he also presented a paper (Johnson 1986). Although creationists did not all abandon the canopy immediately, many creationist organisations conceded the argument was untenable (Hodge 2009). Nevertheless, PACEs retained the canopy until 2016. It is absent from the fourth edition.

Some of the space that was devoted to the vapour canopy is now used instead to deny human-caused climate change. The section begins by acknowledging that Earth’s climate has changed: ‘Vastly different weather patterns and climates developed after the Flood … Today, scientists report that Earth’s climates are still changing’ (ACE 2016b, p. 53). It continues:

Scientific data also shows a rise in carbon dioxide levels in Earth’s atmosphere, but attempts to show a connection between rising global temperatures and rising carbon dioxide levels have failed.

The responses to climate change reports vary greatly. Even though no connection has been made, some still fear that as carbon dioxide levels increase, temperatures will also increase, causing polar ice caps and glaciers to melt … However, as believers … we need not worry that God will lose control of His Creation. God gave us this wonderful promise, 'While the earth remaineth, seedtime and harvest, and cold and heat, and summer and winter and day and night shall not cease.'

(ACE 2016b, p. 53)

The text goes on to say that God is preparing a new Heaven and a new Earth, with a climate far better than any currently known.

In *Science 1096* (ACE 2016c), the final PACE of the eighth grade, ACE students are introduced to evolution for the first time. On its first appearance, evolution is defined as ‘the false theory that assumes that all forms of life developed from lower forms of life’ (ACE 2016c, p. 3). This PACE makes 52 separate factual claims which are said either to cast doubt on evolution or support creationism; 39 of these are repeated from the third edition. Of the other 13, none will be new to those familiar with creationist arguments. Some of them previously appeared in other PACEs, such as the claim that the Mount St. Helens eruption demonstrates that vast geological changes can happen quickly. Others are minor variations on a theme. The third edition claimed that the odds of a human cell evolving are equal to those of ‘one hundred trillion, trillion, trillion, TRILLION blind men’ solving Rubik’s cubes simultaneously (ACE 1986c, p. 3). The fourth edition instead uses the well-worn claim that the odds are like those of a tornado in a junkyard creating a Boeing 747 (ACE 2016c, p. 4).

Only two arguments in the new edition are materially different from those found in previous PACEs. One is the claim that polonium halos found in granite are evidence of a young Earth, a view critiqued by Wakefield (1988) and Baillieul (2005) among others. The other is the claim that traces of blood vessels and soft tissues found in some dinosaur fossils prove that they must have died comparatively recently (ACE 2016c, p. 31). The PACE does not refer to the scientists’ explanations for these findings (Schweitzer, Zheng, Cleland, Goodwin, Boatman, Theil, Marcus and Fakra 2014), nor to the fact that Schweitzer is a Christian who has rebuffed creationist attempts to appropriate her work (Ruppel 2014). The text goes on to argue:

DNA has been found in fossils that some secular scientists claim are millions of years old … Scientists were surprised to find DNA in these ‘old’ fossils and were even more surprised to discover that the DNA was similar to modern DNA. If DNA has been constantly evolving over millions of years, its chemical makeup should be much different from modern DNA, but it is not.

(ACE 2016c, pp. 31–32)

The lack of detail about what fossils were found, when, or by whom makes this claim difficult to check. The most plausible candidate is DNA from fossil magnolia leaves (e.g. Kim, Soltis, Soltis and Suh 2004). This has been touted by Creation Ministries International (Wieland, n.d.), a possible source for ACE’s authors. It remains controversial whether DNA extracted from such samples is authentic or a contaminant (Hebsgaard, Phillips and Willerslev 2005). In any case, the researchers in fact described the lack of DNA sequence divergence between *Magnolia latehensis* and extant species as ‘not surprising’ (Kim, Soltis, Soltis and Suh 2004, p. 617).

The PACE text seems undecided on the degree to which belief in creationism requires faith. At first, it says ‘since science cannot prove the evolutionary model or the Biblical Creation model, each model must be accepted by faith’ (ACE 2016c, p. 5). Later, however, we find ‘little faith is required to believe in a great worldwide Flood … The evidence presented in these exhibits should convince anyone of the truth of God’s Word’ (p. 44). By the end, the text claims we have ‘unquestionable proofs, and a host of indisputable evidence all around us!’ (p. 52).

A number of arguments employed in the third edition have been abandoned, having become untenable even in creationist circles. ACE used to claim that the sun was shrinking at a steady rate, which if projected back over millions of years would mean that it would have swallowed Earth’s orbit. ‘The nuclear fusion theory of how the sun emits heat and light is an invention of evolution scientists’, insisted the text (ACE 1986c, p. 7). ACE still relies on this argument in a staff training PACE in print at the time of writing (ACE 2011, p. 9), which means that staff and students are currently taught directly contradictory statements as the fourth edition science PACE accepts solar fusion without complaint, instead maintaining that the faint young Sun paradox is an insurmountable problem for evolutionists (ACE 2016c, p. 22).

Many other arguments ACE has abandoned work on a similar principle to that of the shrinking sun: assuming a constant rate of change, then multiplying it back over millions of years to produce an absurd conclusion. Using this method, the third edition claimed Earth’s rotation, ocean salinity, dust on the Moon’s surface, the amount of nickel in the ocean, the amount of helium in the atmosphere, the size of the Mississippi delta and the rate of mountain erosion all demonstrated that Earth is young. None is present in the newest PACE. In all, 16 arguments have been dropped. We have no way of knowing for sure why such changes have been made but the fact that more arguments have been removed than have been added perhaps suggests that science communicators have had some success in combating creationist misinformation. Another, related, possibility is that ACE’s changes may be part of the creationism movement’s ongoing, somewhat rearguard, efforts to establish and maintain credibility.

ACE does still point to two lines of evidence that even many creationists regard as discredited: sightings of Noah’s Ark and fossils on the Paluxy River bed in Texas that are claimed to show human footprints alongside dinosaur tracks. Even Answers in Genesis advises against using the latter claim (Mitchell 2012). Despite this, ACE appears unwilling to relinquish this argument. The new text does acknowledge that ‘some Creation scientists now advise against using the prints still remaining in the Paluxy River valley as evidence concerning mankind and dinosaurs’ (ACE 2016c, p. 39). Nonetheless, the tracks are submitted as evidence humans and dinosaurs co-existed. The text then adds for good measure:

However, regardless of these particular footprints, we understand from the Bible that man and dinosaurs were created on the sixth day of Creation and did indeed inhabit and walk on Earth at the same time.

(ACE 2016c, pp. 39–40)

A similar rhetorical strategy is employed with regard to Noah’s Ark. The third edition stated unequivocally ‘The Ark has been seen on many occasions!’ (ACE 1986c, p. 22), and included a photograph captioned ‘Timber from the Ark’ (p. 23). The fourth edition downgrades these to ‘possible’ sightings, before stating ‘as Believers, we do not need to see the Ark to believe it existed. Our faith rests securely in the Bible’ (ACE 2016c, p. 38). Perhaps, some independent-minded students will notice that this cannot claim to be science, even by ACE’s definition of the term.

### Ninth grade

The ninth grade science PACEs constitute ACE’s only high school biology course. The third edition made frequent references to evolution, but the reviewed fourth edition PACEs do not mention it once. There is not though a reduced emphasis on creationism: phrases such as ‘God designed’ and ‘God created’ are abundant. The text does not acknowledge the existence of any alternative viewpoint. One workbook contains sections titled ‘Principles of Inheritance’, ‘Human Genetics’ and ‘Applied Genetics’ (ACE 2020b, pp. 41, 51, 53) but covers these topics without reference to evolution or genetic mutations.

By removing all references to evolution, ACE has made these workbooks less open to ridicule than previous editions, which appealed to the existence of the Loch Ness monster (Scaramanga 2017) as disconfirming evidence of evolution. They still give a misleading impression of science, however. ACE presents biology with no overarching framework for students to understand or connect given facts. At high school level, teaching science exclusively as the memorisation of isolated facts is particularly inadequate. Because these workbooks reject the scientific theory underpinning biology, it is not possible to learn from them a conceptual understanding of biology as a whole, even should an analytically minded student attempt to do so.

Climate change is discussed in the fourth edition, and the tone is again denialist: ‘Objective climate scientists say there is no need to panic about climate change … scientists do not know for certain what is causing these slight temperature changes’ (ACE 2020c, p. 53). The presence of anthropogenic climate change denial alongside creationism is unsurprising. The National Center for Science Education has declared climate change a ‘second front’ alongside evolution in the war on science education because of the frequency with which the two are targeted together (Branch 2013). Creationism is linked to other conspiracy theories by a shared reliance on teleological thinking (Wagner-Egger, Delouvée, Gauvrit and Dieguez 2018).

There is a growing literature on conspiracy theories. Douglas, Uscinski, Sutton, Cichocka, Nefes, Ang and Deravi (2019) argued that they result from a range of psychological, political and social factors. They point out that conspiracy belief is stronger among people who consistently seek patterns and meaning in their environment; it is therefore perhaps unsurprising that they are found among those with fundamentalist beliefs, for whom all that happens is the result of either God’s or the devil’s (or demons’) actions. There is also evidence that conspiracy theories allow people to feel that they are in possession of rare, important information that other people do not have, making them feel special and thus boosting their self-esteem' (Douglas, Uscinski, Sutton, Cichocka, Nefes, Ang and Deravi 2019, p. 9). Belief in conspiracy theories can result in negative societal outcomes by reducing trust between strangers, and within-group cooperation, and increasing prejudice, intergroup conflict, polarisation and extremism (Van Prooijen, Spadaro and Wang 2022).

### Where is the controversy?

Advocates of creationism in the classroom often argue that they want to ‘teach the controversy’ (Scott and Branch 2003). Creationist education, it is said, gives students the opportunity to think for themselves and choose between two sides of the argument. In the PACEs examined, this is manifestly not the case. No account of evolutionary theory is ever offered—students are told the minimum about evolution required to make sense of ACE’s counterclaims. In defending creationism, the PACEs attack abiogenesis, biology, cosmology and geology indiscriminately, without clearly explaining which of these is included in ‘evolution’. A further obstacle is that the PACE authors themselves demonstrate only a limited grasp of evolution. The text talks of a process of evolving into ‘higher’ life forms (ACE 2016c, p. 6). Further misrepresentations of evolutionary theory include:

[I]f fish evolved into frogs, fish should no longer exist, but obviously they do.

(ACE, 2016c, p. 15)

In evolutionary circles, a bird is considered more complex than an octopus. Yet, the eye of an octopus is much more complex than the eye of a bird. If a bird evolved from the octopus, why does the octopus have a more complex eye?

(Ibid, p. 17)

Students are told flatly:

In order for evolution to take place, the DNA of an organism would have to mutate and pass on a trait that was not contained in its original genetic code, which is impossible.

(Ibid, p. 15)

The PACEs misrepresent evidence and present a misleading straw version of evolution, which they pronounce ‘impossible’. In the process, they also present a misleading view of how science works. The conclusion is that evolution is absurd and the only reason anyone believes it is a steely determination to reject God:

A person who is not right with God must find reason, or justification, for not believing. So he readily accepts the theory of evolution, even if many of its arguments are indefensible … In fact, the theory of evolution is a mental justification for unbelief. If unbelievers can accept a theory that leaves God out of the explanation of the origin of the universe, they can live as they please without being morally responsible to their Creator.

(ACE 2016c, p. 52)

The language in the fourth edition PACEs is slightly more temperate, but the conclusion is still clear: those who accept evolution make an immoral choice, and their belief is driven by a determination to sin and to rebel against God. Since the PACEs present evolution as discredited, the implication is that those who believe it are irrational or stupid. The presentation of non-creationists as morally inferior anti-Christians can only promote intolerance and suspicion towards adherents of mainstream science.

In the reviewed workbooks, it is only in the eighth grade that students encounter any exercises whose answer is not found verbatim in the text. These exercises are sufficiently unusual that students are warned ‘The answer to the following question may not be obvious from the text’. Even these are in a multiple choice format and have one acceptable response, for example:

If science and the Bible both support a young Earth and a great worldwide Flood, why do some people still refuse to believe?

- They have not seen enough evidence.
- They have chosen to reject God and His Word.
- They are true scholars.
- They have no interest in science.

(ACE 2016c, p. 53; correct answer is ‘b’)

## Discussion and conclusion

Accelerated Christian Education materials matter to science educators because they are used by quite large numbers of students and present a misleading view of science. Some of the worst examples have been removed in the most recent, fourth, edition of the science PACEs, but overall the fourth edition PACEs are very similar to those in the third edition. The second grade PACEs examined contain 124 pages between them. Of these, 58 are identical in both editions, and a further seven differ by exactly one word. Of 100 activities on the summative ‘PACE tests’, 83 are identical, and eight of those that differ do so by just one word.

The similarities between the other grades are harder to quantify, as much of the text has been reworded while retaining essentially the same meaning. Nevertheless, the large majority of content from the third edition has been retained in the fourth. Evolution is still presented as absurd and discredited, and its adherents considered immoral and foolish. This continues to be taught through a system of rote memorisation via workbooks completed in carrels.

Speck and Prideaux’s (1993) critique that PACEs rely on low-level cognitive tasks is equally applicable to editions released more than two decades later. A more recent study concluded that the third edition ACE science tests were poor quality and unsuitable as evidence of preparation for university (Scaramanga and Reiss 2017). It also found that 100% of ACE science test items in the eighth grade required only simple recall. The PACE tests examined here are similar to those in the third edition and support the earlier findings.

In ACE, we observe an unusual example of evolutionary stasis. The curriculum has remained largely unchanged even though it appears that selection pressures have operated on it. Clearly, the PACE authors are, by and large, little persuaded by the weight of scientific evidence. ACE has also been relatively unmoved by legal challenges: in its first 20 years, ACE was involved in more than 150 lawsuits, most of them relating to accreditation (R. Hunter 1993, p. 171), and online searches reveal subsequent court cases. ACE’s position is that Christian schools should not be regulated in any way, and they and the schools that use its curriculum have used litigation to defend this belief in the USA and elsewhere.

An ACE education is one that materially disadvantages students’ chances of scientific success. It not only puts them at ‘cognitive and conceptual disadvantage’ (Speck and Prideaux 1993, p. 293), but encourages them to develop an intolerant and narrow view of the world. At best, it seems likely that students would emerge quite confused about what evolution actually entails. The PACEs also promote confusion about the nature and methods of science. The individualised workbook format gives students minimal experience of the social process of ‘doing’ science, and the dogmatic tone is not conducive to understanding the ways in which scientific knowledge grows. It is a core aspect of science that any theory, however well established, can be overturned by new evidence. In the PACEs, science is reduced to immediate observation and experiment, a process of straightforwardly ‘proving’ and ‘disproving’ hypotheses, in a way that makes difficult an appreciation of how scientific evidence is used and how theories are formed.

It might be argued that the PACEs could be used as just part of a student’s science curriculum, and a rounded education provided by supplementing ACE with other materials. A number of factors militate against the feasibility of such a solution. ACE schools sign a service agreement committing them to use the curriculum exclusively and according to procedures set out by ACE (2010a, p. 3; 2012, p. 3). Factual distortions in the PACEs would, in any case, limit their usefulness even as supplementary materials. It would also be difficult to use PACEs as a vehicle to encourage critical thinking when the PACE activities each have only one acceptable answer, and the text derides those with different beliefs.

Speck and Prideaux (1993) argued that there is a case for state intervention to protect students in ACE schools. What has subsequently become clear is that ACE is not amenable to changing more than minimally, if at all, its content or methods at the recommendation of those outside its own fundamentalist community. If science education in ACE schools is to improve more than marginally, it is likely only to be through external regulation. We recognise, as did Speck and Prideaux, that state intervention is problematic, particularly where questions of religious liberty arise. Nevertheless, the child’s interests in receiving a sound education, alongside the state’s interest in an educated populace, carry enough weight to make a case for closer state oversight of such schools.

Students who have been taught creationism as science at school seem less likely to choose and succeed at university not only in obviously relevant disciplines such as biology and geology, but also in many other disciplines that are informed by evolution, radiometric dating and/or deep time: archaeology, medicine, agriculture, psychology, linguistics, ancient history and mainstream biblical studies among them. Students educated in this way have diminished opportunities for educational and personal achievement. There may also be a range of costs to wider society. ACE students are likely to need remedial education before pursuing some avenues of academic study or employment. A body of students who have been taught hostility to non-Christians may also threaten a pluralist society (Paterson 2003). Students who are taught that there is no evidence of human-caused climate change are also unlikely to recognise the severity of the present climate crisis (cf. Harrod and Rolland 2021).

It has been argued that creationism is better thought of as a worldview than as a misconception (Reiss 2009). The empirical links between a denial of climate change and a denial of evolution are recognised (Carter and Wiles 2014), and there is a growing literature on the links between political and religious conservatism and cognitive bias (Watts 2017). Students emerging from ACE are likely to have substantial misconceptions about worldviews other than their own. It does not help students from conservative Christian families to leave school believing evolution is simply a fairy tale for atheists. Some emerging from ACE schools later come to feel angry that they were let down by their education (Scaramanga 2017). It should not be the case that children born to creationist parents are doomed to an inadequate science education.

At heart, both climate denialism and creationism are conspiracy theories. When pressed to explain the overwhelming scientific consensus, both the creationist and the climate denier must resort to an imagined cover-up by scientists. If accepted as true, such conspiracy theories undermine the authority of scientific institutions and bodies which accept scientific findings. When only held by a few people, these beliefs may limit adherents’ ability to participate in wider society. If such conspiracy theories are widely accepted, however, they threaten the basis for the production of shared knowledge and the legitimacy of public institutions. Believers in another conspiracy theory, COVID-19 denialism, have shown how severe the consequences of such thinking can be for public life. Given that belief in one conspiracy theory tends to correlate with acceptance of others (Swami, Coles, Stieger, Pietschnig, Furnham, Rehim and Voracek 2011), we should guard against forms of ‘education’ that promote such thinking.

It would help students who have received an ACE education but then moved to mainstream education if their science teachers appreciated not only that they are likely to have been taught very little about the content of evolution but also that they are likely to have been taught a lopsided view as to how science is undertaken, and to have been taught that the theory of evolution is not just mistaken but in contravention of God’s teaching and thus harmful. There is some evidence at undergraduate level that respectful, rigorous and patient teaching about evolution can help creationist students to become more accepting of it (Winslow et al. 2011; Truong, Barnes and Brownell 2018).

Limitations of our study include the fact that we only looked at the third and fourth editions of ACE’s materials and that we sampled from them. Examination of all of ACE’s extant materials from the first edition to the present day would allow a more rigorous analysis of changes. We also restricted ourselves to what might be termed the intended curriculum. There would be considerable value in seeing how these science materials are used in practice. However, this option is likely only to be open to those who are sympathetic to ACE’s practices. We are both known, especially the first author, not to be, so this option was not open to us.

Finally, we believe that our work opens up a number of avenues for future research. Ethnographic work in the sorts of schools that use ACE materials is challenging, principally due to problems over access (Peshkin 1986), but always valuable. Future work could also connect to fears and concerns raised about the home-schooling movement and, particularly in the US, to connections to Christian Nationalism. We have written elsewhere about how the ACE curriculum deals with race (Scaramanga and Reiss 2018), but future research could investigate the possibility of heteronormativity and sexism in ACE curricula and schools that use ACE materials.

## References

[CR1] Abd-El-Khalick F, Lederman NG (2000). The influence of history of science courses on students’ views of nature of science. Journal of Research in Science Teaching.

[CR2] ACE (1986). Science 1086.

[CR3] ACE (1986). Science 1089.

[CR4] ACE (1986). Science 1096.

[CR5] ACE (2010). Procedures manual I: Learning center essentials.

[CR6] ACE (2010). Science 1015.

[CR7] ACE (2010). Science 1017.

[CR8] ACE (2010). Science 1045.

[CR9] ACE (2010). Science 1046.

[CR10] ACE (2010). Science 1047.

[CR11] ACE (2010). Science 1048.

[CR12] ACE (2011). Wisdom.

[CR13] ACE (2012). Administration Manual.

[CR14] ACE (2016). Science 1086.

[CR15] ACE (2016). Science 1089.

[CR16] ACE (2016). Science 1096.

[CR17] ACE. (2019). *A.C.E. curriculum: Scope and sequence.* ACE. Retrieved from https://www.aceministries.com/media/pageimg/Home_Educators_Scope_and_Sequence-2019.pdf.

[CR18] ACE. (2020a). *Global contacts.* Retrieved from https://www.aceministries.com/global-contacts. Archived from the original at http://web.archive.org/web/2020a0607161753/https://www.aceministries.com/global-contacts.

[CR19] ACE (2020). Science 1107.

[CR20] ACE (2020). Science 1108.

[CR21] ACE. (2021). *The Great Commandment and the Great Commission: God’s mandate for Christian education*. ACE. Retrieved from https://www.aceministries.com/media/pageimg/Great_Command_Commission-web.pdf.

[CR22] Alberta Department of Education. (1985). *An audit of selected private school programs: Accelerated Christian Education, Alpha Omega, Mennonite schools, Seventh-Day Adventist schools, and Abeka instructional resources.* Alberta Dept. of Education.

[CR23] Baillieul, T. A. (2005). *“Polonium haloes” refuted.* Retrieved from http://www.talkorigins.org/faqs/po-halos/gentry.html.

[CR24] Baumgardt, J. (2006). *Perceptions of the accelerated Christian education programme as preparation for tertiary education*. Master’s Dissertation. University of South Africa.

[CR25] Beeke, J. W. (1992). *An evaluation of the Accelerated Christian Education (A.C.E.) program for the purpose of Group I Independent School Classification: Summary report*. Ministry of Education, Independent Schools Branch, British Columbia.

[CR26] Berliner DC (1997). Educational psychology meets the Christian Right: Differing views of children, schooling, teaching and learning. Teachers College Record.

[CR27] Beyer CJ, Delgado C, Davis EA, Krajcik J (2009). Investigating teacher learning supports in high school biology curricular programs to inform the design of educative curriculum materials. Journal of Research in Science Teaching.

[CR28] Blancke S, Boudry M, Braeckman J, De Smedt J, De Cruz H (2011). Dealing with creationist challenges. What European biology teachers might expect in the classroom. Journal of Biological Education.

[CR29] Blancke S, Hjermitslev HH, Kjærgaard PC (2014). Creationism in Europe.

[CR30] Branch G (2013). Defending science education: Climate as a second front for biologists. BioScience.

[CR31] Carins, K. (2002). *Graduates’ perceptions of the ACE program as preparation for lifelong learning*. BA dissertation. University of Tasmania.

[CR32] Carter, B. E., & Wiles, J. R. (2014). Scientific consensus and social controversy: Exploring relationships between students’ conceptions of the nature of science, biological evolution, and global climate change. *Evolution: Education and Outreach*, *7*(6). 10.1186/s12052-014-0006-3.

[CR33] ChristianBook.com. (2021a). *4th edition science PACE 1025, Grade 3 Product Slideshow*. Retrieved from https://www.christianbook.com/Christian/Books/product_slideshow?sku=409025&actual_sku=409025&slide=1.

[CR34] ChristianBook.com. (2021b). *Science PACE 1002, Grade 1, 4th edition Product Slideshow*. Retrieved from https://www.christianbook.com/Christian/Books/product_slideshow?sku=6013455&actual_sku=6013455&slide=1.

[CR35] Douglas KM, Uscinski JE, Sutton RM, Cichocka A, Nefes T, Ang CS, Deravi F (2019). Understanding conspiracy theories. Political Psychology.

[CR36] Drisko J, Maschi T (2015). Content analysis.

[CR37] Fairclough N (2003). Analysing discourse: Textual analysis for social research.

[CR38] Fleming DB, Hunt TC (1987). The world as seen by students in Accelerated Christian Education. Phi Delta Kappan.

[CR39] Guhin J (2016). Why worry about evolution? Boundaries, practices, and moral salience in Sunni and Evangelical high schools. Sociological Theory.

[CR40] Guhin J (2021). Agents of God: Boundaries and authority in Muslim and Christian schools.

[CR41] Harms U, Reiss MJ (2019). Evolution education re-considered: Understanding what works.

[CR42] Harrod SE, Rolland V (2021). Factors associated with attitudes and knowledge of first-semester college students toward climate change. BioScience.

[CR43] Hebsgaard MB, Phillips MJ, Willerslev E (2005). Geologically ancient DNA: Fact or artefact?. Trends in Microbiology.

[CR44] Hickman, H., & Porfilio, B. J. (Eds.) (2012). *The new politics of the textbook: Critical analysis in the core content areas*. Sense.

[CR45] Hill BV, Francis L, Thatcher A (1990). Is it time we deschooled Christianity?. Christian perspectives for education.

[CR46] Hodge, B. (2009). *The collapse of the canopy model.* Retrieved from https://answersingenesis.org/environmental-science/the-collapse-of-the-canopy-model/.

[CR47] Hsieh HF, Shannon SE (2005). Three approaches to qualitative content analysis. Qualitative Health Research.

[CR48] Hunter, D. (2014a). *Adventures in ACE V: Senseless about St. Helens.* Retrieved from https://the-orbit.net/entequilaesverdad/2014a/02/26/adventures-in-ace-v-senseless-about-st-helens/.

[CR49] Hunter, D. (2014b). *Adventures in ACE VI: Vacuous about volcanoes.* Retrieved from https://the-orbit.net/entequilaesverdad/2014b/03/06/adventures-in-ace-vi-vacuous-about-volcanoes/.

[CR50] Hunter, D. (2014c). *Adventures in ACE VII: Ignorant about igneous*. Retrieved from https://the-orbit.net/entequilaesverdad/2014c/03/13/adventures-in-ace-vii-ignorant-about-igneous/.

[CR51] Hunter, R. (1985). *Accelerated Christian Education and Church:State relationships in education: A case study analysis* (unpublished EdD dissertation). Southern Illinois University.

[CR52] Hunter, R. (1993). Christian fundamentalist education: A twentieth anniversary. In B. Gilling (Ed.), *Godly schools? Some approaches to Christian education in New Zealand*. University of Waikato.

[CR53] Johnson, R. E. (1986). Perpetuation of creationism through theistic education. In *The proceedings of the first international conference on creationism* (pp. 163–165). Creation Science Fellowship.

[CR54] Katz, I. (2014). *Newsnight*. BBC2.

[CR55] Kim S, Soltis DE, Soltis PS, Suh Y (2004). DNA sequences from Miocene fossils: An *ndhF* sequence of *Magnolia latahensis* (Magnoliaceae) and an *rbcL* sequence of *Persea pseudocarolinensis* (Lauraceae). American Journal of Botany.

[CR56] Klein, R. (2017). Voucher schools championed by Betsy DeVos can teach whatever they want. Turns out they teach lies. *Huffpost*, 15 January. Retrieved from https://www.huffingtonpost.co.uk/entry/school-voucher-evangelical-education-betsy-devos_n_5a021962e4b04e96f0c6093c.

[CR57] Klein, R. (2021). These textbooks in thousands of K-12 schools echo Trump’s talking points. *Huffpost*, 7 December. Retrieved from https://www.huffingtonpost.co.uk/entry/christian-textbooks-trump-capitol-riot_n_6000bce3c5b62c0057bb711f.

[CR58] Laats A (2010). Forging a fundamentalist “one best system”: Struggles over curriculum and educational philosophy for Christian Day Schools, 1970–1989. History of Education Quarterly.

[CR59] Laats A, Angulo AJ (2016). Religion. Miseducation: A history of ignorance-making in America and abroad.

[CR60] Laats A (2021). An eternal monkey trial? Evolution and creationism in US schools. Phi Delta Kappan.

[CR61] Lewis, P. S. (1991). *Private education and the subcultures of dissent: Alternative/free schools (1965–1975) and Christian fundamentalist schools (1965–1990).* PhD thesis. Stanford University.

[CR62] Lombrozo T, Thanukos A, Weisberg M (2008). The importance of understanding the Nature of Science for accepting evolution. Evolution: Education and Outreach.

[CR63] Long D (2011). Evolution and religion in American education: An ethnography.

[CR64] Loxton, R. (2012). How American fundamentalist schools are using Nessie to disprove evolution. *Herald Scotland*. Retrieved from http://www.heraldscotland.com/news/education/how-american-fundamentalist-schools-are-using-nessie-to-disprove-evolution.17918511.

[CR65] McCulloch G (2004). Documentary research in education, history and the social sciences.

[CR66] Matthews, M. R. (2012). Changing the focus: From Nature of Science (NOS) to Features of Science (FOS). In: Khine M. (Ed.) *Advances in Nature of Science research* (pp. 3–26). Springer. 10.1007/978-94-007-2457-0_1.

[CR67] Miller JD, Scott EC, Okamoto S (2006). Public acceptance of evolution. Science.

[CR68] Miller JD, Scott EC, Ackerman MS, Laspra B, Branch G, Polino C, Huffaker JS (2022). Public acceptance of evolution in the United States, 1985–2020. Public Understanding of Science.

[CR69] Mitchell, E. (2012). *Paluxy River tracks in Texas spotlight.* Retrieved from https://answersingenesis.org/dinosaurs/footprints/paluxy-river-tracks-in-texas-spotlight/.

[CR70] Moore R, Jones L, Reiss M (2007). The history of the creationism/evolution controversy and likely future developments. Teaching about scientific origins: Taking account of creationism.

[CR71] Moser C, Mueller D (1980). Accelerated or exaggerated? An evaluation of Accelerated Christian Education. Lutheran Education.

[CR72] Newall, E. (2021). *Evolution and the controversy: Existential and psychoanalytic perspectives.* EdD thesis. University College London.

[CR73] Nordin VD, Turner WL (1980). More than segregation academies: The growing Protestant fundamentalist schools. The Phi Delta Kappan.

[CR74] Paterson, F. R. A. (2003). *Democracy and intolerance: Christian school curricula, school choice, and public policy*. Phi Delta Kappan.

[CR75] Peshkin A (1986). God’s choice: The total world of a fundamentalist Christian school.

[CR76] Reiss MJ (2009). The relationship between evolutionary biology and religion. Evolution.

[CR77] Reiss MJ (2011). How should creationism and intelligent design be dealt with in the classroom?. Journal of Philosophy of Education.

[CR78] Reiss, M. J. (2018) Creationism and Intelligent Design. In P. Smeyers (Ed.), *International handbook of philosophy of education* (pp. 1247–1259). Springer. 10.1007/978-3-319-72761-5_86.

[CR79] Rose SD (1988). Keeping them out of the hands of Satan: Evangelical schooling in America.

[CR80] Ruppel, E. (2014). *Not so dry bones: An interview with Mary Schweitzer.* Retrieved from http://biologos.org/blogs/archive/not-so-dry-bones-an-interview-with-mary-schweitzer.

[CR81] Scaramanga, J. (2017). *Systems of indoctrination: Accelerated Christian Education in England.* PhD thesis. University College London.

[CR82] Scaramanga J, Reiss MJ (2017). The suitability of the International Certificate of Christian Education as an examination for university entrance. Oxford Review of Education.

[CR83] Scaramanga J, Reiss MJ (2018). Accelerated Christian Education: A case study of the use of race in voucher-funded private Christian schools. Journal of Curriculum Studies.

[CR84] Schweitzer MH, Zheng W, Cleland TP, Goodwin MB, Boatman E, Theil E, Marcus MA, Fakra SC (2014). A role for iron and oxygen chemistry in preserving soft tissues, cells and molecules from deep time. Proceedings of the Royal Society B: Biological Sciences.

[CR85] Scott EC, Branch G (2003). Evolution: What’s wrong with “teaching the controversy”. Trends in Ecology & Evolution.

[CR86] Shaw, M. (2009). Fundamentalist exams on a par with A-level. *Times Education Supplement*, p. 1.

[CR87] Speck C, Prideaux D (1993). Fundamentalist education and creation science. Australian Journal of Education.

[CR88] Stern F, Kampourakis K, Huneault C, Silveira P, Müller A (2018). Undergraduate biology students’ teleological and essentialist misconceptions. Education Sciences.

[CR89] Swami V, Coles R, Stieger S, Pietschnig J, Furnham A, Rehim S, Voracek M (2011). Conspiracist ideation in Britain and Austria: Evidence of a monological belief system and associations between individual psychological differences and real-world and fictitious conspiracy theories. British Journal of Psychology.

[CR90] Truong JM, Barnes ME, Brownell SE (2018). Can six minutes of culturally competent evolution education reduce students’ level of perceived conflict between evolution and religion?. The American Biology Teacher.

[CR91] Van Prooijen JW, Spadaro G, Wang H (2022). Suspicion of institutions: How distrust and conspiracy theories deteriorate social relationships. Current Opinion in Psychology.

[CR92] Vardiman, L. (1986). The sky has fallen. In *The proceedings of the first international conference on creationism* (pp. 113–119). Creation Science Fellowship.

[CR94] Wagner-Egger P, Delouvée S, Gauvrit N, Dieguez S (2018). Creationism and conspiracism share a common teleological bias. Current Biology.

[CR95] Wakefield JR (1988). The geology of Gentry’s “Tiny Mystery”. Journal of Geological Education.

[CR96] Watts F (2017). Psychology, religion, and spirituality: Concepts and applications.

[CR97] Whitcomb JC, Morris HM (1961). The Genesis flood: The Biblical record and its scientific implications.

[CR98] Wieland, C. (n.d.). DNA dating: Positive evidence that the fossils are young. *creation.com*. Retrieved from http://creation.com/dna-dating-positive-evidence-that-the-fossils-are-young.

[CR100] Winslow MW, Staver JR, Scharmann LC (2011). Evolution and personal religious belief: Christian university biology-related majors’ search for reconciliation. Journal of Research in Science Teaching.

